# Autoimmune Diseases and the Vestibular and Oculomotor System: Clinical Presentation, Diagnosis, and Treatment

**DOI:** 10.3390/jemr19040071

**Published:** 2026-07-02

**Authors:** Felix K. Schwarz, Gerald Wiest, Paulus Rommer

**Affiliations:** 1 Department of Neurology, Medical University of Vienna, 1090 Vienna, Austria; 2 Comprehensive Center for Clinical Neurosciences and Mental Health, Medical University of Vienna, 1090 Vienna, Austria

**Keywords:** oculomotor, vertigo, autoimmune

## Abstract

Vertigo, dizziness, and oculomotor disturbances may occur as manifestations of immune-mediated disorders affecting the inner ear, central vestibular pathways, or multisystem autoimmune disease. Although uncommon, these conditions are clinically important because delayed recognition may lead to irreversible hearing loss, vestibular dysfunction, or neurological disability. This review summarizes the clinical presentation, diagnostic approach, and treatment of immune-mediated vestibular and oculomotor disorders. We suggest a practical classification into isolated immune-mediated inner ear disease, systemic autoimmune disorders with audio-vestibular involvement, and autoimmune disorders of the central or peripheral nervous system affecting balance and eye movements. Red flags for such conditions include bilateral or progressive symptoms, fluctuating audio-vestibular deficits, associated neurological signs, and accompanied autoimmune disease. Corticosteroids remain the main first-line treatment in many of these disorders, mainly due to missing data from controlled trials. Steroid-sparing immunosuppressants, biologics, and tumor-directed therapies are effective in many cases; however, because of the missing data, they are only used in selected entities without any other choice. A structured neuro-otological and immunological workup is essential to improve diagnostic accuracy and enable timely therapy.

## 1. Introduction

Vertigo and dizziness are frequent but often underdiagnosed symptoms in autoimmune disorders. In some patients, they are the first sign of disease and may be accompanied by other audio-vestibular symptoms [1,2]. Vertigo can also be associated with objective oculomotor signs, such as nystagmus and other involuntary eye movements. These findings help distinguish whether the problem is arising from the peripheral vestibular system or from central vestibular pathways. Autoimmune-mediated dizziness usually results from an inappropriate immune response against an autoantigen. This immune activation may be triggered by abnormal antigen expression on cell surfaces, as in paraneoplastic syndromes, or by immune-cell infiltration into previously protected tissues after trauma or secondary inflammation [3,4]. Clinically, autoimmune vestibular disorders are often difficult to recognize early. This makes timely diagnosis important, because early treatment may reduce the risk of irreversible damage.

Autoimmune vestibular disorders can be divided into two main patterns: those that primarily involve the inner ear and those associated with systemic autoimmune disease. Systemic cases can be further separated into disorders that directly target the inner ear and those that affect the central vestibular pathways. Ralli et al. proposed three major groups for the classification of autoimmune vestibular disorders [5]:

- Isolated immune-mediated inner ear disorders;
- Systemic autoimmune diseases with audio-vestibular involvement;
- Autoimmune disorders of the nervous system that affect the balance system.

The inner ear contains the sensory organs for both hearing and balance, which share the same hair cells. These mechanoreceptors convert motion into chemical signals through the neurotransmitter glutamate and are highly sensitive to injury or environmental change, including inflammation [5].

The vestibular system, including the saccule, utricle, and semicircular canals, detects head position and movement and supports the vestibular-ocular reflex. They are part of the endolymphatic system, a system now recognized as immunocompetent, with both cellular and humoral immune response capabilities [6,7]. Here, immune-mediated dysfunction may cause spontaneous nystagmus, abnormal head-impulse findings, and other signs of peripheral vestibular failure. Peripheral vestibular nystagmus is clearly characterized and may occur as either a paretic or irritative nystagmus with consistent features [8,9].

By contrast, central vestibular disorders involve the brainstem and cerebellar networks that integrate sensory input from the eyes, inner ear, and peripheral nerves. Damage in these regions can produce heterogeneous nystagmus patterns, as well as abnormalities of saccades, smooth pursuit, gaze stabilization, and gaze holding deviating from peripheral vestibular patterns [10].

Disruption at either level can lead to ocular motor disturbances such as nystagmus, diplopia, or ocular deviation, which may in turn produce vertigo [11]. 

Because the inner ear and central nervous system have limited regenerative capacity, early recognition and treatment are especially important.

Although most patients with vertigo or dizziness have benign, vascular, or functional causes, certain findings should raise suspicion for an immune-mediated disorder. These red flags include bilateral symptoms, subacute or rapidly progressive hearing loss, fluctuating audio-vestibular deficits, accompanying ocular inflammation, additional neurologic or systemic signs, atypical nystagmus patterns, and a poor fit with common vestibular syndromes. A structured diagnostic workup should include bedside vestibular and oculomotor examination, audiometry, vestibular testing, brain MRI with attention to the inner ear and brainstem, and selected serological or cerebrospinal fluid studies depending on the suspected phenotype.

After infectious disease has been excluded, corticosteroids are used in most cases of autoimmune-mediated audio-vestibular disease. Early treatment is associated with better symptom response and less long-term disability, so prompt recognition is critical [12].

### 1.1. Anatomy and Physiology of the Vestibulo-Oculomotor System

The peripheral vestibular system is part of the inner ear. It detects head movement and position so the brain can maintain posture, balance, and stable vision. It consists of three semicircular canals and two otolith organs, the utricle and saccule, in each ear. The semicircular canals detect angular acceleration of the head, whereas the otolith organs are sensitive to horizontal and vertical acceleration. This information is sent through the vestibular nerve to the brainstem and cerebellum. The vestibular-ocular reflex (VOR) is a reflex that stabilizes gaze by moving the eyes in the opposite direction of head movement.

Pathological, involuntary eye movements in the peripheral vestibular disorders discussed below result from an imbalance of the VOR. The VOR is a reflex system capable of keeping the fovea stable during movements of the head and body. When this system is damaged, patients can develop oscillopsia and/or peripheral vestibular nystagmus [13]. Peripheral vestibular nystagmus arises due to a one-sided tonic loss of function of the VOR, with reduced excitation of the extraocular muscles on the contralateral side and a resulting drift of the eyes toward the less active side. Following the deviation, a rapid corrective saccade then brings the eyes back, producing the fast phase of nystagmus [14]. According to Alexander’s law, the fast phase of peripheral vestibular nystagmus beats away from the side with reduced function (paretic nystagmus). During fixation, the slow-phase velocity (SPV) of peripheral vestibular spontaneous nystagmus is usually diminished; on the other hand, it increases when the fixation is removed (i.e., under Frenzel Glasses).

Oscillopsia is the sensation that the visual world is moving or unstable during walking or head movements, resulting from a weakened vestibular-ocular reflex (VOR). While vertigo is typically associated with an acute, one-sided vestibular lesion, oscillopsia more often reflects a bilateral VOR deficit [15] so that it is absent when the head and body are held still. The problem appears only during motion, when the impaired VOR fails to stabilize gaze, creating the perception of a “bouncing” or unstable environment.

The central vestibular system comprises the brainstem and cerebellum, which receive, integrate, and modulate afferent signals from the peripheral vestibular apparatus to support gaze stabilization, postural control, and spatial orientation, with central pathways extending all the way to the vestibular cortex. Within the brainstem, the vestibular nucleus complex serves as the central connection, integrating semicircular canal and otolith input with visual, proprioceptive, and cerebellar information. Cerebellar structures, particularly the vestibulocerebellum (flocculus, nodulus, and uvula), control and modify central oculomotor functions, including velocity storage mechanisms and vestibular-ocular reflex suppression.

A key mechanism behind central vestibular nystagmus is a disturbance of the velocity storage system. This neural network prolongs and spatially organizes vestibular signals beyond the brief input coming from the peripheral vestibular apparatus. It relies mainly on the vestibular nuclei and is strongly influenced by the cerebellar nodulus and uvula [16,17]. Lesions in these regions can lead to central vestibular spontaneous nystagmus (SPN) in horizontal, vertical, or torsional directions, as well as head-shaking nystagmus (HSN) that appears in a different plane than the one tested (cross-coupled HSN), and direction-changing nystagmus [17]. In addition, it can lead to ocular tilt and impaired visual vertical perception [18].

An important central oculomotor function is gaze holding, mediated through the neural integrator. The neural integrator, which includes the horizontal and vertical gaze centers, transforms brief eye velocity commands into sustained signals that keep the eyes in an eccentric position. When this mechanism is impaired, gaze holding becomes “leaky,” and, clinically, this appears as gaze-evoked nystagmus (GEN), with the eyes drifting back toward the center and being corrected by recurrent saccades [19].

VOR suppression is a physiological mechanism that inhibits the vestibular-ocular reflex when the head and fixation target move together in the same direction, enabling stable visual fixation on objects during head movements. Physiologically, this inhibition occurs through vestibulocerebellar circuits, particularly the flocculus and nodulus of the cerebellum, which integrate vestibular signals with oculomotor pursuit signals and reduce VOR gain. With intact VOR suppression, the eyes remain stable relative to the orbit during rotation because the reflexive counter-rotation mechanism is suppressed. Pathologically, dysfunction of vestibulocerebellar pathways leads to incomplete VOR suppression, manifesting as saccadic intrusions and eye-tracking movements during head rotation. VOR suppression deficits are characteristic of central vestibular pathologies, particularly cerebellar lesions, and can occur in age-associated balance disorders with increased fall risk. Clinically, the VOR suppression test serves as an important diagnostic tool for distinguishing between peripheral and central vestibular disorders [20].

Saccades are rapid, jerky eye movements that deliberately align gaze toward new fixation targets, enabling quick vision switches from one point to another [21]. They occur both voluntarily (for example, during reading) and reflexively in response to sudden stimuli and are essential for the visual exploration of the environment. Smooth pursuit, by contrast, consists of slow, continuous eye movements that track a moving object so that its image stays on the fovea and remains clear [22]. These two eye movement systems serve different purposes, depend on largely distinct neural circuits, and together support accurate and efficient visual perception of both static and moving targets.

### 1.2. Vestibular Functional Testing

Regarding vestibular function testing, the caloric test of the vestibular apparatus involves irrigating the external ear canal with warm or cold water, which creates a temperature gradient that moves the endolymph in the horizontal semicircular canal and triggers the vestibular-ocular reflex. The video-head-impulse test (vHIT) uses infrared tracking of the pupils to measure VOR gain in all three planes, enabling the examiner to test all six semicircular canals selectively. Another difference is the spectrum of VOR testing, as caloric testing assesses low-frequency deficits, while vHIT uses high acceleration to test for high-frequency responses of the VOR [23]. Vestibular-evoked myogenic potentials (VEMPs) can assess the function of the sacculus and utriculus, which are not evaluated via either caloric testing or vHIT, and help complement the examination of the peripheral vestibular organ [24].

Video-oculography (VOG) is a method for recording and quantifying eye movements using infrared video goggles to track the pupil position and motion of both eyes simultaneously. Eye movements can be assessed both during visual fixation and with fixation removed. Using VOG, nystagmus can be characterized by measuring slow-phase velocity (SPV), and saccadic performance can be evaluated for velocity, as well as hypo- or hypermetria, alongside the accuracy and gain of smooth pursuit. VOG findings are interpreted in conjunction with rotational chair testing to assess VOR gain and suppression as well as to analyze post-rotatory eye movements.

### 1.3. Pathophysiological Mechanisms of Autoimmune Diseases

Autoimmunity develops when immune tolerance to self-antigens fails, so that immune responses are misdirected against the body’s own tissues, producing chronic inflammation and tissue damage. This loss of self-tolerance reflects a multifactorial interaction of genetic susceptibility, environmental influences, and disturbances in immune regulatory pathways.

Autoimmune diseases, and the underlying immune dysregulation, can be organized according to their dominant effector mechanisms, recognizing that most disorders combine both cellular (T-cell-mediated) and humoral (B cell/autoantibody-mediated) components. The Gell and Coombs hypersensitivity framework, originally proposed for allergic reactions, is often applied to autoimmunity, with types II, III, and IV corresponding to three major pathogenic patterns seen in many autoimmune conditions.

Type II (antibody-mediated) hypersensitivity is characterized by autoantibodies binding directly to cell surface molecules or receptors, leading to functional disturbance or complement-dependent cytotoxicity, as exemplified by antibodies against NMDA receptors, LGI1, or CASPR2. Type III (immune complex-mediated) hypersensitivity centers on the formation of antigen–antibody complexes in the circulation, which lodge in vessel walls, engage Fc receptors on leukocytes, and trigger acute inflammatory vasculitis. Type IV (T-cell-mediated) hypersensitivity involves autoreactive T lymphocytes that cause tissue injury through direct cytotoxicity or the activation of macrophages and other effector cells.

Identifying the predominant hypersensitivity mechanism has direct therapeutic implications. Disorders primarily driven by type IV mechanisms can respond favorably to treatments targeting T-cell activation or trafficking, such as agents blocking T-cell adhesion (e.g., natalizumab). Type III-dominated diseases often benefit from strategies that decrease immune complex burden and complement activation. In type II-mediated diseases, interventions like plasmapheresis to remove pathogenic antibodies or B-cell-depleting therapies can be particularly effective.

## 2. Autoimmune Vestibular Disorders

### 2.1. Isolated Immune-Mediated Inner Ear Diseases

The precise etiology of autoimmune-mediated inner ear disease remains poorly understood. Traditionally, the inner ear was thought to be an immunologically privileged site, largely because of the absence of conventional lymphatic drainage and the protection of the highly selective blood-labyrinth barrier [25]. For many years, the endolymphatic sac—an anatomically secluded extension of the endolymphatic duct—was regarded as the sole immunocompetent compartment of the inner ear, containing resident leukocytes and controlling local antibody production [26]. However, more recent investigations have demonstrated the presence of immune cells in additional inner ear structures, including the cochlea and vestibular labyrinth, suggesting a broader distribution of immunological activity within the inner ear than previously recognized [27,28]. Although not definitively established, immune mechanisms are suspected to contribute to some of the following disorders.

The detection of both autoantibodies [29] and pro-inflammatory cytokines [30,31] indicates that these disorders involve contributions from both the adaptive and innate immune system. These immune responses can affect all endolymphatic spaces of the cochlea, vestibule, and semicircular canals [32]. The primary clinical manifestation presents as rapidly progressive bilateral hearing loss, vertigo, or dizziness and tinnitus depending on the specific autoimmune disease.

#### 2.1.1. Autoimmune Inner Ear Disease (AIED)

Autoimmune inner ear disease (AIED) is a rare clinical entity characterized by bilateral sensorineural hearing loss (SNHL). It is estimated to account for less than 1% of all SNHL cases [33], with variable findings concerning sex distribution. Some studies found higher prevalence in women [3,5] while others could not find a gender predilection [34]. AIED may present as an isolated inner ear disorder. However, a recent study found an association with a systemic autoimmune disease in 16% of cases [33].

##### Clinical Presentation

The SNHL in AIED predominantly affects the high-frequency range, with a threshold reduction of at least 30 dB. Onset is usually rapidly progressive, developing over the course of weeks to months, which distinguishes it from sudden SNHL (onset within <72 h) and presbycusis (gradually progressive). In early stages, unilateral involvement is common, and clinical manifestations can be highly variable. Vestibular symptoms, such as episodic vertigo, are reported in approximately 50% of patients. Additional symptoms may include aural fullness and tinnitus [35]. Bilateral occurrence and the rapid SNHL make it distinguishable from the “classical” form of Ménière’s disease (MD); however, overlaps with the autoimmune subgroup of MD make it difficult to diagnose, as treatment for MD remains mostly symptomatic in contrast to AIED, for which fast corticosteroid treatment can prevent lasting damage [36].

##### Pathophysiology

Unlike other autoimmune diseases, no specific autoantibody has yet been identified that would enable diagnosis or serve as a therapeutic target. A recent review discussed several serum biomarkers, concluding that the detection of inner-ear-specific biomarkers in peripheral blood is a possibility in cases with disrupted brain–blood barrier; however, evidence for the relevance of these biomarkers remains insufficient for clinical practice [37]. One reason for a missing diagnostic marker might be the sheer number of possible biomarkers and the heterogeneity of their appearance. For instance, high-anti-choline titers have been observed in groups of patients with AIED, supporting the assumption of a cochlear-specific antibody immune response (Gell and Coombs type II), while other AIED patients have shown only very low titers or other inconsistent antibodies [38].

Furthermore, there is evidence of T-cell-driven interleukin (IL)-17 and Interferon-gamma secretion in patients with AIED (Gell and Coombs type IV) [38]. Additionally, the involvement of the innate immune system, in terms of resident macrophages of the inner ear, has been discussed as well [4]. Cytokines apart from (IL)-17 also appear to play a central role in AIED and have been investigated in both human and animal studies. Tumor necrosis factor (TNF)-alpha appears to be the most important cytokine here, followed by IL-1 and IL-6 [38]. Similarly to other autoimmune diseases, such as rheumatoid arthritis, AIED exhibits characteristics of both immune-mediated and autoinflammatory diseases [39].

Animal experiments on the key limpet hemocyanin (KLH) have described the abovementioned immune reactions [40], but their results have been of limited use for therapeutic interventions as the reactions could not be reproduced in humans [38]. Experimental evidence from a murine model demonstrated autoimmune-mediated destruction of cochlear and vestibular structures without the presence of endolymphatic hydrops, suggesting that hydrops is not a necessary pathological mechanism in AIED. Instead, tissue injury appears related to autoimmune targeting of type II collagen and inflammatory damage within the organ of Corti and vestibular apparatus (Gell and Coombs type III) [2].

In conclusion, the pathophysiology of AIED remains unclear, and type II, III, and IV autoimmune reactions have been discussed [4]. However, available research is limited because (1) access to the cochlea is restricted and (2) peripheral blood tests may not provide meaningful insight into processes in the inner ear.

At present, there is no disease-specific therapy, and systemic corticosteroids remain the mainstay of treatment in the early stages, with immunosuppressive therapies and biologics following in the later stages or in cases with poor response [41].

##### Diagnosis

There is no definitive test for AIED; rather, it is a diagnosis of exclusion. Clinicians should suspect AIED when patients present with rapidly progressive (typically evolving over weeks) sensorineural hearing loss. Diagnostic evaluation includes thorough clinical assessment, laboratory testing, consideration of proposed but non-validated serological markers such as anti-myelin P0, anti-β-actin, anti-cochlin, and anti-KHRI-3 antibodies, and assessment of the clinical response to immunosuppressive therapy [42]. In addition, diagnostic criteria (Table 1) have been proposed [43]. Furthermore, brain magnetic resonance imaging (MRI) is essential to exclude other etiologies, and rheumatologic evaluation is recommended given the association of AIED with systemic autoimmune conditions, such as rheumatoid arthritis (RA), or a Brain–Ear–Eye Syndrome (BEE), as systemic involvement is identified in approximately 15% of cases [33]. MRI of the temporal bone can help differentiate AIED from chronic otitis media with SNHL [44]. Other apparative diagnostics such as vHIT, calorics, and VEMPs can unmask vestibular involvement and may help distinguish AIED from the main differential diagnosis of MD.

##### Differential Diagnosis

Given the fluctuating nature of sensorineural hearing loss, along with associated symptoms such as vertigo, tinnitus, and aural fullness, MD is the primary differential diagnosis, especially in the early stages of AIED. As mentioned above, a murine model showed autoimmune-mediated damage to the inner ear in the absence of endolymphatic hydrops, which, if proven right, might help in differentiating AIED from MD.

Additional differential considerations include ototoxicity secondary to pharmacologic agents, otosyphilis, toxoplasmosis, large vestibular aqueduct syndrome, and intracranial hypertension.

##### Treatment

AIED is rare, with about 5–20 cases of SNHL per 100,000 patients, but it is also one of the few forms of SNHL that may be reversible with prompt and appropriate treatment. Systemic corticosteroids remain the first-line therapy and are most effective when initiated early in the disease course. In cases of secondary AIED associated with systemic autoimmune disorders, treatment regimens may require adjustment or extension to address the underlying systemic pathology. Second-line immunosuppressive agents, including methotrexate, cyclophosphamide, and azathioprine, have been employed with variable efficacy and are often limited by their adverse effects. Biologic therapies are currently under investigation for their potential role in refractory cases [12].

#### 2.1.2. Ménière’s Disease (MD)

Ménière’s disease (MD) is an idiopathic inner ear disorder that presents with sensorineural hearing loss (SNHL), intermittent episodes of vertigo, and tinnitus [45,46]. Initially, there may only be vertigo and a feeling of pressure on the affected ear, and the typical hearing loss may not occur until later in the course. Involvement is often unilateral; in up to 40% of cases, both sides may be affected. MD can occur at any age, but it most commonly affects patients between the ages of 20 and 40. Recently, differentiation of MD into five subtypes was proposed with one “classical” and four atypical phenotypes [45]. The atypical forms include delayed endolymphatic hydrops-like phenotypes, genetic forms, migraine-associated variants, and autoimmune subtypes. In this review, we will mainly focus on the autoimmune subtype.

##### Clinical Presentation

The classic symptom triad consists of SNHL, which is mainly found in the low- to mid-frequency range, intermittent vertigo, and low-pitched roaring tinnitus [45]. Vertigo attacks usually present as spinning attacks lasting 20 min up to 12 h, or up to 24 h in rare cases. In addition, patients often report a sensation of auricular pressure or fullness. During the attacks, spontaneous irritative nystagmus can be observed [47,48] beating towards the affected side. After many years of illness, a spontaneous paretic nystagmus, beating away from the side of the lesion, can sometimes also be observed in interictal periods [49]. The symptoms accumulate and intensify episodically, and while the vertigo usually resolves, the hearing loss and tinnitus may persist after the episodes [46].

##### Pathophysiology

The histopathological correlate of MD is endolymphatic hydrops (EH), a pathological dilatation of the endolymphatic spaces of the labyrinth [46]. Although this is found in all patients with MD, not all patients with endolymphatic hydrops are symptomatic. Thus, the relevance of hydrops to symptomatology has not been established with certainty. The pathophysiology of the development of hydrops (and symptoms) has not been conclusively determined. Multiple mechanisms and factors have been proposed in the development of MD, including allergic, traumatic, infectious, genetic, and possible immune-associated contributions [45,50]. An immune-associated hypothesis has been proposed only for a subset of patients, mainly based on the co-occurrence of systemic autoimmune diseases [45,51], but also in context with bilateral occurrence and response to corticosteroids [12,45]. However, there is no evidence for a generalized autoimmune origin of MD.

Within the immune-mediated hypothesis, there are various theories regarding the contribution of the immune system to the pathophysiology of the MD autoimmune subgroup (aMD). All of them are based on a triggering event (e.g., an infection or trauma), which in turn leads to an immune reaction via either cytokine release [52] and macrophage activation [53] (Gell and Coombs type IV) or, as in Guillain–Barré syndrome, to inner ear damage through a cross-reaction of antibodies (Gell and Coombs type II) [3]. Due to the anatomically isolated (“immune privileged”) localization of the inner ear, the immune system may be unaware of all antigens the inner ear contains—in the sense of immunologically sequestered or “privileged” antigens—and, after initial contact following infection or injury, it may recognize newly presented inner ear antigens as foreign [25].

Within the autoimmune subgroup, further distinctions can be made, as different levels of interleukins or antibodies can be measured. The first, second, and third groups showed high levels of tumor necrosis factor alpha, elevated IgE levels, and increased levels of IL-1β, respectively [54,55].

Recent studies have also suggested a possible role of autoantibodies against thyroid antigens, like those in Hashimoto’s thyroiditis and Graves’ disease, in inner ear structures [12]. This is supported by the observation that a significantly increased number of thyroid antibodies was found in MD patients [56]. However, elevated levels of thyroid autoantibodies have also been found in benign paroxysmal positional vertigo (BPPV) [57].

##### Diagnosis

There is no single confirmatory diagnostic test. A combination of history and clinical presentation is suggestive, but as with AIED, it is a diagnosis of exclusion. Standard diagnostic tests include audiometry, electronystagmography, rotational testing, and imaging of the cerebellopontine angle. Concerning vestibular testing, a combination of caloric testing and vHIT might be helpful. Historically, a common dissociation was normal vHIT with abnormal caloric responses. This was not true in a comparative study, where out of 88 patients with definite MD, 67% (*n* = 59) showed abnormal caloric testing; however, of these patients with abnormal caloric testing, only 45% had normal horizontal vHIT. However, when including the findings of the vertical canals, this number dropped to 18% [58].

Hydrops-MRI involves capturing a delayed gadolinium-enhanced image of the inner ear, with endolymphatic hydrops showing on the affected side. This technique can point strongly towards MD diagnosis. Inflammation appearing as perilymphatic enhancement on MRI has been shown to develop into an endolymphatic hydrops. This observation could be used in the absence of EH to help diagnose MD [55,59].

In 2015, the Bárány Society defined criteria to aid in the diagnosis of Ménière’s disease (Table 2) [60].

##### Differential Diagnosis

The main differential diagnoses are vestibular migraine, BPPV, vestibular schwannoma, perilymph fistula, and vestibular neuritis [60]. Patient history can be helpful in differentiating between AIED and MD, as the latter is considered an episodic disease with relapsing symptoms in the early course of the disease [46] and the former shows continuous and progressive symptoms.

##### Treatment

Treatment aims to improve the symptoms, i.e., minimize the frequency of vertigo attacks, reduce tinnitus, and prevent progressive hearing loss. Dietary measures and medical therapy are commonly used and may improve symptoms in a substantial proportion of patients. Betahistine is the drug of choice for treating vertigo [46], while corticosteroids can be used for refractory symptoms. Evidence for corticosteroids is limited, showing heterogeneous results for systemic use, with benefits in cases of intrathympanic application. There are also positive data on gentamicin instillations to positively influence vertigo attacks [61], and some studies have shown promising effects on the auditory symptoms in patients treated with osmotic diuretics, especially glycerol [62,63]. However, a systematic review with 848 patients showed only low certainty for diuretics [64].

#### 2.1.3. Bilateral Vestibulopathy (BVP)

Bilateral Vestibulopathy (BVP) is a condition comprising inner ear disorders characterized by the failure or dysfunction of both labyrinths and vestibular nerves. The defining feature of all these diseases is the bilaterally reduced or even absent VOR function. Causes include ototoxicity and meningitis, as well as peripheral vestibular causes such as MD or genetic variants in cases of CANVAS (cerebellar ataxia, neuropathy, vestibular areflexia syndrome). However, a definite cause is rarely found or can only be assumed based on patient history.

##### Clinical Presentation

Clinically, the patient presents with motion-dependent episodes of vertigo, which may be accompanied by hearing loss, as well as gait and stance unsteadiness. In the early stages, rotational vertigo is also possible due to acute vestibular damage. The symptoms worsen in the dark or on uneven ground (e.g., when standing on a cushion) and typically resolve when the patient is sitting or lying down [3]. Patients might describe oscillopsia, a visual disturbance characterized by the sensation of environmental motion. This symptom arises from damage to the vestibular organs, leading to a deficit of the VOR. As a result, the image of the environment cannot be held stable on the retina during movement, resulting in image motion on the retina (“retinal slip”).

##### Pathophysiology

Bilateral Vestibulopathy results from bilateral dysfunction of the vestibular end organs or vestibular nerves. Recognized causes include ototoxicity (e.g., Amiodaron, Cisplatin, Gentamycin), infections (e.g., meningitis), Ménière’s disease, and genetic factors; however, in approximately 50–70% of cases, the cause remains unexplained [65,66,67]. In addition, in some cases, immune-mediated mechanisms have been proposed, as autoimmune diseases have been reported in up to 25% of patients. This is supported by findings of serum IgG reactivity against labyrinthine structures, including the ampullae, semicircular canals, saccule, and utricle [67,68]. Although immune-mediated BVP is a rare form of an already rare disease, therapeutic implications arise from the diagnosis and should therefore be considered and investigated.

##### Diagnosis

Due to its commonly heterogeneous presentation, low prevalence, and slow progression of symptoms, a diagnosis of BVP is often delayed. Diagnosis is based on clinical history in combination with bedside examination and instrument-based vestibular assessment, including balance testing, head impulse testing, and video-oculography with rotational tests. Typically, a bilaterally impaired or completely absent vestibulo-ocular reflex (VOR) is found [69], and complementary MRI of the brain, auditory-evoked potentials, and rheumatologic workup are useful. In addition, diagnostic criteria have been defined (Table 3) [69].

##### Differential Diagnosis

Important differential diagnoses include CANVAS, cerebellar ataxias, ototoxic vestibulopathy, bilateral Ménière’s disease, and central causes of gait instability.

##### Treatment

Treatment depends on the underlying cause of BVP. In selected cases, with laboratory and radiological evidence of immune-mediated etiology, corticosteroids may be considered, and vestibular rehabilitation remains a key component of management [67].

### 2.2. Systemic Autoimmune Diseases with Audio-Vestibular Involvement

The separation between isolated inner ear involvement and systemic autoimmune diseases is subtle. The following is a brief overview of autoimmune diseases in which the inner ear can be primarily affected, leading to peripheral and central vestibular and oculomotor symptoms.

As these diseases are rare and often heterogeneous and relapsing-remitting, symptoms are often not linked to each other. However, in contrast to isolated immune-associated inner ear diseases, both etiology and pathophysiology are better understood, and diagnostic markers are partially available. These diseases are grouped together as Brain–Eye–Ear (BEE) Syndromes.

BEE Syndrome: Audio-vestibular, ocular, and central nervous symptoms are common manifestations of autoimmune diseases [70]. Autoimmune diseases in which this triad is common include Susac syndrome, Cogan syndrome, Vogt–Koyanagi–Harada syndrome, systemic lupus erythematosus, Sjögren’s syndrome, Behçet’s disease, and Sarcoidosis. Similarly to Ménière’s disease, the triad can evolve over the course of the disease. At onset, only one isolated symptom can be present, posing a diagnostic challenge [70].

In these diseases, central vestibular and oculomotor pathways may be involved. This widens the spectrum of pathological eye movements. Apart from central nystagmus, i.e., spontaneous nystagmus (SPN) in contrast to Alexander’s law, gaze-evoked nystagmus (GEN), or purely vertical and/or torsional nystagmus, saccadic intrusions and deficits of the VOR suppression may be observed.

Collectively, a study found nystagmus of peripheral and central vestibular origin in 30 out of 90 patients with systemic autoimmune diseases with audio-vestibular involvement [71].

#### 2.2.1. Susac Syndrome

##### Clinical Features

Susac syndrome is a rare organ-specific autoimmune endotheliopathy affecting the microvascular endothelium of the brain, retina, and inner ear. It occurs mainly in young women. The classic triad of symptoms consists of encephalopathy, retinal vascular occlusions with scotomas, and low-frequency bilateral sensorineural hearing loss (SNHL) [72]. Typically, audio-vestibular symptoms such as tinnitus (54%), nystagmus, and vertigo (67%) also occur [73]. Nystagmus can arise from canal paresis as a peripheral vestibular nystagmus or from lesions of the brainstem and cerebellum, leading to GEN or central positional nystagmus [74,75].

##### Diagnosis

The exact pathophysiology is not yet known; however, recent studies suggest the involvement of both antibody-mediated (Gell and Coombs type II) and cellular immune mechanisms (Gell and Coombs type IV). On the one hand, anti-endothelial cell antibodies (AECA) have been detected in approximately 25% of patients [72]; on the other hand, natalizumab, an autoantibody preventing T-cell migration, has been shown to be effective [76]. In the diagnosis of Susac syndrome, three apparative methods play an important role in addition to the clinical findings: fluorescence angiography (FA), MRI, and audiometry. MRI reveals pathognomonic “snowball lesions” in the corpus callosum; however, lesions to the brainstem occur in up to 30% of patients, affecting the medial longitudinal fasciculus (MLF), cerebellar peduncles, and the cerebellum itself [74].

##### Treatment

There is no standardized therapy [72,77]. However, early and aggressive corticosteroid therapy alone or in combination with intravenous administration of immunoglobulins is reported to be effective in the literature [78,79]. In the absence of a response, second-line therapy with rituximab, cyclophosphamide, or azathioprine may be administered [80].

#### 2.2.2. Cogan Syndrome (CS)

##### Clinical Features

Cogan syndrome is a rare autoimmune disease associated with ocular and audio-vestibular manifestations [81]. The disease occurs predominantly in young adults and is characterized by non-syphilitic interstitial keratitis and Ménière-like symptoms with unilateral or bilateral sensorineural hearing loss (SNHL), dizziness, and tinnitus [82]. Hearing loss usually presents with rapid progression (1–3 months). Nystagmus can be found in 20% of patients, most notably peripheral vestibular nystagmus, followed by positional nystagmus and GEN [5,83]. Systemic vasculitis can be seen in about 10% of cases, leading to affection of the aortic valve as well as the coronary arteries, kidney vessels, or arteries supplying blood to the brain. Typically, there is less than 2 years between the onset of ocular and audio-vestibular changes [84].

##### Diagnosis

Pathophysiologically, it is assumed that CS is an autoimmune/inflammatory disease. This theory is supported by the fact that autoantibodies can be detected in many cases against inner ear antigens and corneal structures (so-called Cogan peptides), as well as anti-neutrophil cytoplasmic antibodies (Gell and Coombs type II/III) [82]. However, the antibodies are non-specific, and to date, no confirmatory diagnostic test has been established [85]. Therefore, it is a diagnosis of exclusion. In this case, it is difficult to distinguish the disease from Ménière’s disease, especially in atypical courses and/or in the absence of ocular symptoms.

##### Treatment

Corticosteroids are the treatment of choice, although TNF-alpha blockers such as Infliximab have also shown promising results in small case series, particularly for audio-vestibular symptoms [82,86]. Therapy-resistant progressive hearing loss may require cochlear implantation.

#### 2.2.3. Vogt–Koyanagi–Harada Disease

##### Clinical Features

This is a rare multisystemic granulomatous autoimmune disease that manifests primarily in melanocyte-rich tissues such as skin, inner ear, meninges, and hair [87]. Asian, Hispanic, and Native American populations are more commonly affected, with disease beginning between the ages of 30 and 50. The primary presentation is bilateral chronic diffuse granulomatous panuveitis. After an acute initial stage, systematized vertigo with hearing loss often occurs. Peripheral vestibular nystagmus can be observed; however, if the inflammation spreads to the brainstem or cerebellum, GEN can also be observed [88]. Tinnitus has also been described. Other prodromal symptoms include headache and periocular pain [89] as well as integumentary signs like vitiligo, poliosis, and alopecia [90].

##### Diagnosis

The exact cause of the disease is unclear; however, it is thought to be a CD4+T-cell-mediated immune reaction (Gell and Coombs type IV) against melanocytes with a genetic predisposition [90]. The diagnosis is primarily made based on clinical presentation.

##### Treatment

The treatment of choice is the administration of high-dose corticosteroids as early as possible. With timely treatment, the prognosis is good, as approximately 60% of patients experience a significant improvement in symptoms [91].

#### 2.2.4. Behçet’s Disease

##### Clinical Features

This is a systemic autoimmune disease that manifests histologically as leukocytoclastic vasculitis (Gell and Coombs type III) and can affect various organ systems. Classic symptoms are recurrent oral or genital ulcers, eye inflammation, and skin lesions [12,92], and inner ear involvement has been described in 12–80% and central nervous system (CNS) in up to 10% of cases [5,93], with brainstem/mesencephalic lesions being the most common side. Accordingly, peripheral vestibular nystagmus alongside central vestibular nystagmus may occur. However, third and sixth nerve palsy seem to be much more prevalent, presenting with diplopia and eye deviation (eso- or exophoria) [92].

##### Diagnosis

An exact cause is not known. However, both genetic predisposition and environmental factors appear to play a role in the development of the disease. The presence of HLA-B 51 is considered a significant genetic risk factor. Imaging lesions of Behçet’s disease are mainly found in the area of the mesencephalon [94]. Lesions in this region can affect both oculomotor and central vestibular function, leading to various vertigo symptoms. In up to 80% of cases, SNHL is also described, which often makes differentiating it clinically from Ménière’s disease difficult [5].

##### Treatment

Therapeutically, treatment with corticosteroids, as well as other immunosuppressants, including cyclophosphamide and methotrexate, is recommended. According to recent studies, the neurological symptoms also respond well to TNF-alpha blockers [95] or interleukin-1 inhibitors [96].

#### 2.2.5. Sjögren Syndrome (SS)

##### Clinical Features

Sjögren syndrome is a chronic autoimmune disorder that affects exocrine glands to the point of loss of function due to periductal lymphocytic infiltration (Gell and Coombs type IV) [97]. The disease occurs mainly in females (10:1) and typically begins between the ages of 40 and 50 [70]. SS can also affect the peripheral or central nervous system (CNS). Reports have shown the involvement of the nervous system in up to 70% of patients [98].

CNS involvement seems to be less common than that of the peripheral nervous system (PNS) [99], but can occur simultaneously. Symptoms of PNS affection are similar to other forms of peripheral neuropathy and can result in imbalance. CNS involvement includes focal signs such as motor, sensory, and cerebellar deficits, as well as aphasia, dysarthria, seizures, and movement disorders. However, otological symptoms such as tinnitus, vertigo, and sensorineural hearing loss (SNHL) seem to occur in 22–46% of cases [5,100]. Sjögren Syndrome with CNS involvement manifests histologically as an immune-mediated small-vessel vasculopathy (Gell and Coombs type III), and the theory of the immune-mediated mechanism of CNS involvement is supported by cerebrospinal fluid findings showing intrathecal IgG synthesis in patients with active CNS involvement. It is assumed that the otological symptoms are caused by involvement of the stria vascularis of the basal turn of the cochlea [101]. In a review of patients with cerebellar involvement, nystagmus was found in 15% of patients but was not characterized [102]. GEN and central positional nystagmus have been reported [103]. Positional nystagmus due to peripheral vestibular excitation of a semicircular canal (SCC) occurs in accordance with Ewald’s first law. SCC stimulation results in nystagmus in the same plane as the stimulated canal. If the position and alignment of the semicircular canal do not match the observed nystagmus, central positional nystagmus—due to damage to the afferent otolith pathways of the cerebellum, especially nodulus, uvula, or tonsils—should be considered [104].

##### Diagnosis

Diagnostic criteria for SS are available from the ACR-EULAR classification criteria. Diagnosis requires at least four points. It should be emphasized that, together with a positive salivary gland biopsy, positive anti-SSA/Ro antibodies constitute one of the two main criteria, making it a valuable serum marker [105,106].

##### Treatment

Treatment options for SS consist of symptomatic treatment, in addition to systemic treatment, with immunosuppressive agents like corticosteroids, methotrexate, mycophenolate, azathioprine, and cyclophosphamide and biologics like rituximab being valid options [105,107].

### 2.3. Autoimmune Diseases of the Central and Peripheral Nervous System

Direct involvement of central pathways or the peripheral nervous system due to autoimmune diseases can cause vertigo or dizziness with nystagmus. Sensorineural hearing loss (SNHL) is rare in these diseases and can distinguish them from inner ear involvement. The vestibular findings in these diseases vary, depending on the origin of the disease, with autoimmune encephalitis showing clear central vestibular deficits (mainly cerebellar); multiple sclerosis shows central vestibular deficits; and sarcoidosis and GBS are able to show a mixture of both or either alone.

#### 2.3.1. Multiple Sclerosis (MS)

Vertigo or dizziness is a common symptom of multiple sclerosis that can present as the initial manifestation of the disease or during its course [108]. This is already evident from Charcot’s triad with nystagmus alongside tremor and scanning speech. Up to 5% of patients report dizziness as the symptom that most affects their quality of life.

Different types of acute vertigo can be distinguished: acute vestibular syndrome (AVS) and positional vertigo [109].

AVS is the rapid onset of spontaneous vertigo that lasts for days to weeks (>24 h) and is accompanied by nausea, vomiting, nystagmus, gait unsteadiness, and head movement intolerance [110]. This symptomatology may strongly resemble that of vestibular neuritis; however, AVS in MS is strongly associated with active lesions in the area of the CN VIII, either at the root entry zone or in the area of the medial vestibular nucleus [109]. MRI has shown contrast uptake of the cranial nerves in approximately 8% of MS patients, and the eighth cranial nerve was involved in 1.6% of these cases [111]. Also, some medullar lesions can resemble the symptoms of a vestibular neuritis if vestibular pathways are affected (i.e., Medial longitudinal fascicle (MLF)). MLF lesions are, due to the length of the white matter fascicle, frequent in MS and are classically associated with internuclear ophthalmoplegia (INO) [112], a defined syndrome of impaired contralateral adduction and ipsilateral nystagmoid eye movement. However, depending on the extent of damage, contralesional horizontal gaze palsy may not be present, but pathways of the vestibulo-ocular reflex (VOR) to and from the abducens nucleus may be affected [113,114], leading to oscillopsia and vertigo [113]. Moreover, downbeat nystagmus (DBN) and gaze-evoked nystagmus are common presentations in patients with active cerebellar lesions, and even lasting acquired pendular nystagmus has been described [115,116,117].

In contrast, the symptoms of central positional vertigo present as short-lasting vertigo, triggered by head movement and can be clinically confused with benign paroxysmal positional vertigo.

Positional vertigo is a common symptom in patients with MS. It has been shown that about 52% of patients with MS and vertigo do indeed suffer from BPPV; however, they are often misdiagnosed and unnecessarily treated with corticosteroids under the suspicion of disease activity. Even though BPPV is common in MS patients, not all positional vertigo is due to BPPV. Lesions in the cerebellar peduncle have been associated with central positional nystagmus [104]. Nystagmus can appear as direction-changing in different positions, as DBN, or as persistent positional nystagmus [117], in contrast to classical BPPV.

Vestibular symptoms can be an early sign of a relapse [118]; however, even in the absence of acute vestibular symptoms, vestibular functions can be impaired. Abnormal nystagmography findings in MS patients can often be attributed to lesions in the brainstem area and lead to chronic dizziness.

#### 2.3.2. Autoimmune Encephalitides

Autoimmune encephalitis can present as very heterogeneously. In many cases, however, the first symptoms include vertigo or dizziness, which precedes the diagnosis by up to months [119]. This section focuses on autoantibody-mediated disorders in which the vestibulocerebellar syndrome is the primary or sole clinical symptom.

Autoantibodies involved may be directed against intracellular (Gell and Coombs type IV), cell surface, or synaptic neural antigens (Gell and Coombs type II) [1].

Antibodies against intracellular nuclear and cytoplasmic antigens are generally present in paraneoplastic syndromes. The underlying immunopathogenic process is assumed to be neuronal dysfunction mediated by cytotoxic T cells and the consecutive destruction of cerebellar Purkinje cells, which in turn leads to oculomotor dysfunction [1,120]. The specific contribution of the involved autoantibodies remains unclear. The identification of these types of antibodies should lead to an aggressive and focused search for associated malignancies, most often lung (SCLC), testicular, breast, or ovarian tumors [121]. Treatment of an underlying cancer is the main goal of any therapy. Immunotherapy concerning the activated T cells may relieve symptoms; however, patient outcomes are poor [1].

Conversely, antibodies against extracellular cell surfaces or synaptic antigens are mainly found in non-paraneoplastic autoimmune vestibulocerebellar syndromes, and these seem to be directly involved in immunopathogenesis. Antibody binding is assumed to lead to subsequent functional changes in the ion channels and synaptic neurotransmitter receptors, resulting in neuronal malfunction along the vestibulocerebellar pathways [122]. The association with cancer varies but seems to be much higher in patients with paraneoplastic antibodies [123]. The response to immunotherapy, particularly antibody-depleting treatments, is substantial, and outcomes in these diseases are generally regarded as favorable.

Typical presentation is subacute onset of cerebellar symptoms over the course of weeks to months, consisting of limb or gait ataxia and dysarthria [122]. Oculomotor symptoms may be observed early in the course of the disease, while other cerebellar symptoms may still be absent, and are often missed without a specific vestibulocerebellar examination. Opsoclonus or ocular flutter are one of the most classically associated clinical manifestations for cerebellar paraneoplastic diseases. Both are a special variant of saccadic intrusion, with spontaneous back-to-back saccades without any intersaccadic pauses. The different terminology originates from the fact that ocular flutter only affects horizontal saccades, whereas opsoclonus shows saccades in the horizontal and vertical plane. Both symptoms arise from a cerebellar lesion affecting Purkinje cells, disinhibiting the fastigial nucleus of the cerebellum. The fastigial nucleus is responsible for inhibiting burst neurons of the paramedian pontine reticular formation (PPRF) of the midbrain. Failure to suppress these burst neurons leads to opsoclonus or ocular flutter.

However, other saccadic intrusions, downbeat or gaze-evoked nystagmus, and VOR suppression deficits due to cerebellar degeneration or malfunction can be documented via video-oculography in most cases [120,124]. The presence and degree of other neurologic symptoms such as cognitive impairment, seizures, psychiatric symptoms, neuropathy, movement abnormalities, hearing impairment, and autonomic features vary among the different antibody syndromes.

In particular, recurrent episodes of vertigo and dizziness can occur with regard to Anti-Ri (ANNA-2), GAD65 antibody, VGCC antibody, Anti-DNER (anti-Tr), Neurochondrin antibody, and mGLuR1 [125], while patients suffering from Anti-Yo (PCA-1), PCA-2, Amphiphysin antibody, ITPR1 antibody, Anti-Ca (anti-GRAF), and Septin-5 antibody present with a chronic form of vertigo or dizziness [1].

Kelch-like protein 11 encephalitis seems to be particularly associated strongly with neuro-otologic symptoms, including central vestibular or gaze-evoked nystagmus in up to 60%, gaze palsy in 45% of patients, and deficits in smooth pursuit or saccadic anomalies, among other findings [120,126,127].

#### 2.3.3. Sarcoidosis

Sarcoidosis is an immune-mediated disease associated with granulomatous inflammation of affected organs [128]. Neurosarcoidosis (NS) may affect the central or peripheral nervous system independently or simultaneously and lead to significant morbidity. Sarcoidosis is an inflammatory disease hallmarked by an enhanced cell-mediated granulomatous response (Gell and Coombs type IV) to, currently, unknown antigens [70]. Our current knowledge of the pathogenesis of sarcoidosis is mainly based on studies on pulmonary sarcoidosis. To what degree these findings can be applied to NS and whether the immunology of NS varies from that of sarcoidosis in other organs is the subject of ongoing research. A defining feature of sarcoidosis is the aggregation of granulomas that cause organ dysfunction. Granulomas consist of a variety of immune cells, including epithelioid macrophages and CD4+ T helper cells in the center, surrounded by fibroblasts, B cells, and CD8+ cytotoxic T cells. NS can present with a wide range of neurologic symptoms, but the leading symptoms in NS are cranial neuropathy (up to 50% of cases, mainly affecting VIII CN) and peripheral neuropathy (40% of cases) with a range of large- and small-fiber polyneuropathies and polyradiculoneuropathies [129,130].

Randomized trials to guide the treatment of NS have yet to be conducted. Knowledge about treatment is purely based on case series or expert opinions. Generally, corticosteroids are the treatment of choice in most cases [131].

#### 2.3.4. Guillain–Barré Syndrome/Miller Fisher Syndrome

Acute inflammatory demyelinating polyneuropathy is a postinfectious disease that occurs often in cases of infection with *C. jejuni* and is characterized by an abnormal autoimmune response targeting peripheral nerves and spinal roots. It is often summarized under the term Guillain–Barré syndrome (GBS). Molecular mimicry between microbial antigens and the triggering nerve antigen is the driving force behind the development of this disease (Gell and Coombs type II). First symptoms usually develop 1–2 weeks after initial immune response and can be very subtle in the form of bilateral symmetric limb weakness, sensory deficits, and hyporeflexia.

Several different autoantibodies against neuronal membrane ganglioside are associated with GBS and linked to different subtypes.

Miller Fisher syndrome (MFS) is considered an atypical variant of GBS associated with Anti-GQ1b antibodies (Ab). The clinical triad for MFS consists of cranial neuropathy, ataxia, and areflexia, but does not need to be fully developed [132].

MFS can cause acute unilateral vestibulopathy (AUVP) by involving the peripheral vestibular structures, in cases where cranial neuropathy manifests as an external ophthalmoplegia or affects the vestibulocochlear nerve. However, it has been shown that GQ1b Ab can access the brainstem through a local disruption of the blood–nerve barrier near the roots of the cranial nerves [133] and cause damage to central vestibular structures.

Accordingly, ocular motor findings are heterogeneous. In a case series of three patients, spontaneous horizontal and vertical nystagmus, gaze-evoked nystagmus, head-shaking nystagmus (horizontal and cross-coupled), positional nystagmus, impaired vestibular-ocular reflex, and smooth pursuit were reported [134]. Another study found that in comparison to AUVP without ganglioside Ab, SPN was less intense in patients presenting with anti-GQ1b or anti-GM1 Ab [135]. Two things are noteworthy: First, patients with AVS in the context of MFS showed normal tendon reflexes, even though high concentrations of GQ1b Ab were detected. Second, central vestibular symptoms were reported, even though GBS and its variants are usually diseases of the peripheral nervous system. This is believed to arise from the presence of GQ1b and GM1 receptors in the cerebellum [135]. Treatment with intravenous immunoglobulin or corticosteroids has been proven effective, and full recovery can be expected within 6 months [136].

## 3. Conclusions

**Figure 1:**
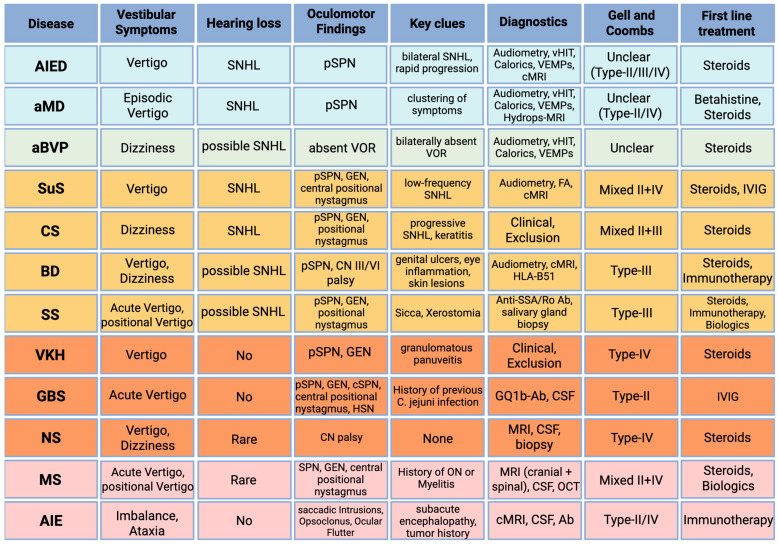
Summary table of autoimmune diseases and their associated vestibular and oculomotor manifestations, together with relevant treatment approaches.

In conclusion, autoimmune mechanisms should be considered an important differential diagnosis for patients presenting with audio-vestibular syndromes (Figure 1). Early recognition is essential, as timely treatment may prevent irreversible damage.

**Figure 2:**
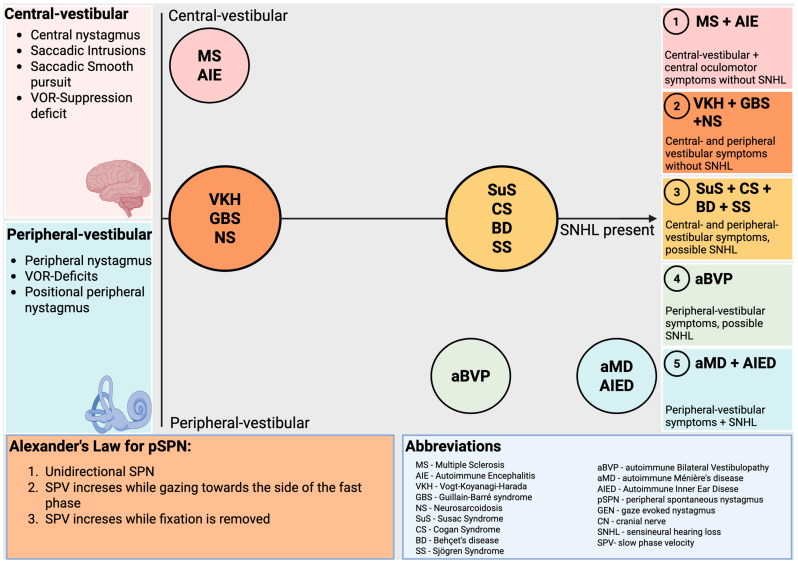
Schematic overview of autoimmune diseases with vestibular and oculomotor involvement, grouped according to central vestibular versus peripheral vestibular presentations and the presence or absence of sensorineural hearing loss (SNHL).

During bedside examination, the origin of the audio-vestibular symptoms needs to be localized, especially through careful assessment of vestibular eye movements and oculomotor function to differentiate peripheral from central vestibular involvement. Unidirectional horizontal nystagmus with preserved central oculomotor function may suggest peripheral pathology, especially in combination with sensorineural hearing loss (SNHL), whereas direction-changing, vertical, or gaze-evoked nystagmus, impaired smooth pursuit, abnormal saccades, or any nystagmus with normal function of the VOR indicates central vestibular involvement (Figure 2).

Once the lesion has been localized, it should be correlated with the patient’s history to determine which of the classic vestibular diagnoses applies. In cases where the test results and the patient’s history do not fit any of the classic diagnoses and additional red flags are present—particularly the occurrence of bilateral or progressive symptoms, fluctuating audio-vestibular deficits, unexplained SNHL, neurological signs, or a personal history of systemic autoimmune disease—further evaluation for an autoimmune cause should be conducted.

Once suspicion is raised, targeted diagnostic evaluation, including laboratory testing for systemic and organ-specific autoimmunity, cerebrospinal fluid analysis, apparative audio-vestibular testing, vestibular function assessment, and MRI-based imaging, should be pursued to identify inflammatory or immune-mediated pathology.

Finally, early initiation of immunotherapy—including corticosteroids and, in selected cases, steroid-sparing immunosuppressive or biologic therapies—remains crucial for the improvement of functional outcomes and for limiting permanent neuro-otologic impairment.

To facilitate clinical recognition of autoimmune audio-vestibular disease, a practical mnemonic-based framework may be useful. One possible acronym is “FLARE,” emphasizing key clinical warning signs: Fluctuating course, Lateralization changes or bilateral involvement, Accompanying neurological or systemic manifestations, Rheumatic or autoimmune background, and Exception to common vestibular patterns.

Such an approach may help clinicians systematically identify patients requiring further autoimmune workup, because despite advances in neuroimmunology, significant gaps remain. There is a pressing need for validated biomarkers, improved antibody stratification, prospective cohort data, and controlled treatment trials specifically addressing audio-vestibular syndromes of autoimmune origin. A deeper understanding of immunopathophysiological mechanisms will likely improve diagnostic precision and enable more targeted therapeutic strategies in the future.

When clarifying symptoms of vertigo and associated oculomotor disorders, an autoimmune genesis should therefore be considered after excluding the most common causes, and appropriate evaluation should be initiated. In particular, in patients with known autoimmune disease and possible audio-vestibular or eye movement involvement must not be overlooked, as early therapy can prevent long-term sequelae.

## Figures and Tables

**Table 1:** Proposed diagnostic criteria of AIED.

Major Criteria: Bilateral SNHL Presence of systemic autoimmune disease Ana-titer > 1:80 Reduced number of naïve T cells (cd4ra) Recovery rate of hearing > 80% after treatment
Minor Criteria: Unilateral SNHL Young/middle-aged patient Female sex Recovery rate of hearing < 80% after treatment
Three major criteria or two major criteria and more than two minor criteria would support the diagnosis of an AIED [43].

**Table 2:** Diagnostic criteria of Ménière’s disease.

Definite Ménière’s Disease >2 Spontaneous episodes of vertigo (20 min–12 h) Low- to medium-frequency sensorineural hearing loss Fluctuating aural symptoms (hearing, tinnitus, or fullness) Not better accounted for by another vestibular diagnosis
These criteria apply to the diagnosis of definite Ménière’s disease. Separate criteria have also been proposed for probable Ménière’s disease [60].

**Table 3:** Diagnostic criteria of bilateral vestibulopathy.

Definite BV: Chronic vestibular syndrome with unsteadiness when walking or standing + either/and Movement-induced blurred vision or oscillopsia during walking or quick head/body movements Worsening of unsteadiness in darkness and/or on uneven ground No symptoms while sitting or lying down under static conditions Bilaterally reduced or absent angular VOR function documented with video-head-impulse test, calorics, or rotatory chair Not better accounted for by another disease
These criteria reference the diagnosis of definite bv; alternatively, probable bv can be diagnosed following a set of criteria [69].

## Data Availability

No new data were created or analyzed in this study. Data sharing is not applicable to this article.

## Abbreviations

The following abbreviations are used in this manuscript:

Ab	Antibodies
AIED	Autoimmune inner ear disease
AUVP	Acute unilateral vestibulopathy
AVS	Acute vestibular syndrome
BEE	Brain–Ear–Eye Syndrome
BPPV	Benign paroxysmal positional vertigo
BVP	Bilateral vestibulopathy
CANVAS	Cerebellar ataxia, neuropathy, vestibulopathy, and areflexia syndrome
CNS	Central nervous system
CS	Cogan syndrome
DBN	Downbeat nystagmus
EH	Endolymphatic hydrops
FA	Fluorescence angiography
GBS	Guillain–Barré syndrome
GEN	Gaze-evoked nystagmus
HSN	Head-shaking nystagmus
IL	Interleukin
INO	Internuclear ophthalmoplegia
KLH	Key limpet hemocyanin
MD	Ménière’s disease
MFS	Miller Fisher syndrome
MLF	Medial longitudinal fascicle
MRI	Magnetic resonance imaging
MS	Multiple sclerosis
NS	Neurosarcoidosis
PNS	Peripheral nervous system
PPRF	Paramedian pontine reticular formation
RA	Rheumatoid arthritis
SCC	Semicircular canal
SNHL	Sensorineural hearing loss
SPN	Spontaneous nystagmus
SPV	Slow-phase velocity
SS	Sjögren syndrome
TNF-alpha	Tumor necrosis factor-alpha
VEMPs	Vestibular evoked myogenic potentials
vHIT	Video-head-impulse test
VOR	Vestibulo-ocular reflex
